# Photoassisted Charging of Li-Ion Oxygen Batteries Using g-C_3_N_4_/rGO Nanocomposite Photocatalysts

**DOI:** 10.1021/acsami.2c05607

**Published:** 2022-07-21

**Authors:** Ersu Lökçü, Nilay Kaçar, Meltem Çayirli, Reşat Can Özden, Mustafa Anik

**Affiliations:** Department of Metallurgical and Materials Engineering, Eskisehir Osmangazi University, 26040 Eskisehir, Turkey

**Keywords:** g-C_3_N_4_/rGO, nanocomposites, photocharging, Li-ion oxygen batteries, photocatalyst

## Abstract

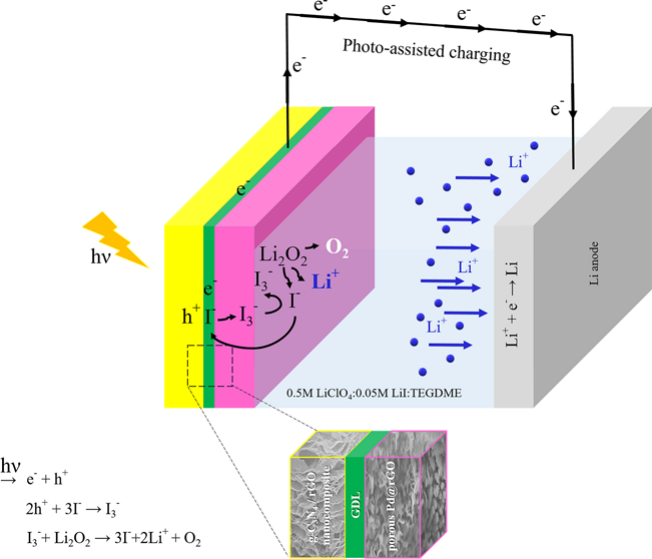

In this work, g-C_3_N_4_/rGO nanocomposites were synthesized to use them as photocatalysts in Li-ion oxygen batteries by aiming at the reduction of the charging potential efficiently under photoassisted conditions. Fourier transform infrared (FTIR) spectra showed that novel C=C bonds formed between g-C_3_N_4_ and rGO during the decomposition of melamine and that the formation of these bonds was assumed to cause a red shift in the optical absorption band edge. The competition between the narrowing in the optical band gaps of the nanocomposites as a result of the red shift due to the presence of rGO and the degradation in the visible light utilization as a result of favorably absorbed incident light by rGO instead of g-C_3_N_4_ pointed out that the g-C_3_N_4_/3% rGO nanocomposite has the optimum light absorbance efficiency. The photoassisted charging tests indicated that the g-C_3_N_4_/3% rGO nanocomposite reduced the charging potential effectively, especially at higher current densities, and improved the cyclic discharge–charge performance of the Li-ion oxygen batteries considerably.

## 1 Introduction

Li-ion oxygen batteries with ∼3560 Wh kg^–1^ theoretical specific capacity have attracted great research attention.^1−3^ One of the focuses of these studies is to reduce the charging potential of the Li-ion oxygen batteries (∼4.2 V_Li^+^/Li_) since the sluggish oxidation kinetics of the low-conducting Li_2_O_2_, which is the main discharge product, causes extended overpotential.^4^ The high charging potential results in not only the low energy efficiency but also the electrochemical decomposition of the active battery components.

Yu et al.^5^ proposed a photoassisted charging of the Li-ion oxygen batteries with the aid of triiodide/iodide (I_3_^–^/I^–^) redox shuttling by integrating photoelectrode (dye-sensitized titanium dioxide) into the battery cell (three-electrode cell: anode, cathode, and photoelectrode) to reduce the charging potential down to the discharging potential levels (∼2.72 V_Li^+^/Li_). The redox shuttling (I_3_^–^/I^–^) between Li_2_O_2_ particles and the oxygen electrode surface facilitates the oxidation of Li_2_O_2_ and reduces the oxidation overpotential gradually (∼3.5 V_Li^+^/Li_) without any illumination.^6^ Upon illumination (the photoassisted charging), the photoexcited electrons of the photoelectrode transfer to the anode to reduce the lithium cations, and the generated photovoltage is utilized in the reduction of the charging potential.^5^ The charging voltage is determined by the difference between the Li^+^/Li redox potential and photoelectrode conduction band (CB) edge potential.^5^ During the photoassisted charging, I^–^ ions are oxidized to I_3_^–^ ions by the photoexcited holes on the photoelectrode and then I_3_^–^ ions diffuse to the oxygen electrode surface (cathode) where they are reduced back to I^–^ ions by oxidizing the Li_2_O_2_, spontaneously.^5^ Liu et al.^7^ took the photoassisted charging process one step further, and instead of integrating the photoelectrode as a third electrode into the Li-ion oxygen battery cell, they loaded g-C_3_N_4_ as the photocatalyst on the carbon paper, and they used carbon paper as both the photoelectrode and cathode. The g-C_3_N_4_ with ∼2.7 eV band gap and 1.7 V_Li^+^/Li_ CB edge potential level provided as low as 1.9 V_Li^+^/Li_ charging potential, which was even lower than the discharging potential.^7^ Obviously, photoassisted charging opens a new pathway to overcome the extended overpotential problem in the Li-ion oxygen batteries.

The performance of the g-C_3_N_4_ as the photocatalyst in the reduction of the charging potential of the Li-ion oxygen battery was extraordinary.^7^ Indeed, g-C_3_N_4_, a nonmetallic semiconductor, has been explored extensively as a visible-light-active photocatalyst since it is well known with a relatively small band gap, cost efficiency, and thermal and chemical stabilities.^8−12^ The poor electrical conductivity and the severe recombination of the photogenerated electron–hole pairs, however, limit the large-scale applications of g-C_3_N_4_.^13^^13^ The synthesis of g-C_3_N_4_-based nanocomposites is accepted as the main strategy to eliminate these handicaps and improve the visible light absorption performance of g-C_3_N_4_.^14−17^ Especially due to the similar carbon network and sp^2^-conjugated π structure, graphene and g-C_3_N_4_ are considered to be the most compatible materials to form nanocomposites.^14,15,18−22^ The reduced graphene oxide (rGO) has an additional advantage over graphene or doped graphene due to the presence of oxygen-rich active sites on it since these active sites result in the formation of novel covalent bonds in the nanocomposites.^14,15,23^ It is reported that the band gap, CB edge potential, and thus the valance band (VB) edge potential of g-C_3_N_4_ can be tuned effectively by intercalation of various amounts of the rGO.^14,15^ More specifically, the narrowed band gap due to the red shift of the absorption band edges, the positively shifted VB edge potential, and the enhanced electronic conductivity cause the improved photocatalytic activity to better utilize visible light and increase the oxidation power upon the synthesis of the g-C_3_N_4_/rGO nanocomposites.^14,15^ The red shift of the absorption band edges was also reported in TiO_2_/rGO nanocomposites.^23^

In this work, g-C_3_N_4_/rGO nanocomposites are synthesized to use as the photoelectrode in the Li-ion oxygen battery, the motivation being the reduction of the charging potential under photoassisted conditions. The improved photocatalytic activity is expected to provide a stable reduced charging potential during the long discharge/charge cycles at especially relatively high current densities that the literature involving the photoassisted charging of the Li-ion oxygen battery lacks these data. The contrary points, in our work, as compared to the procedure described by Liu et al.^7^ can be underlined such that while one of the surfaces of the gas diffusion layer (GDL) was loaded by the g-C_3_N_4_/rGO nanocomposite photocatalyst (photoelectrode), the other surface of the GDL was loaded by the porous Pd@rGO (cathode; oxygen electrode). The aim of our work was not only to achieve a photoelectrode with improved photocatalytic activity but also to achieve a cathode with an increased surface area, which is very critical for the oxygen electrode.

## 2 Experimental Section

### 2.1 Materials

Graphite flakes (100 mesh, ≥75% min), sulfuric acid (H_2_SO_4_, 98%), phosphoric acid (H_3_PO_4_, 85%), potassium permanganate (KMnO_4_, 99%), hydrogen peroxide (H_2_O_2_, 35%), hydrochloric acid (HCl, 37%), *N*-methyl-2-pyrrolidone (NMP, C_5_H_9_NO, anhydrous, 99.5%), ethanol (C_2_H_5_OH, ≥99.9%), methanol (CH_3_OH, ≥99.9%) tetraethyl orthosilicate (Si(OC_2_H_5_)_4_, 98%), Pluronic F108 (∼14 600, PEG–PPG–PEG), dimethoxydimethylsilane (DMDMS, 95%), ammonia solution (NH_4_OH, 25%), ethylene glycol (C_2_H_6_O_2_, 99.8%, anhydrous), potassium chloride (KCl), dipotassium hydrogen phosphate (K_2_HPO_4_, anhydrous), lithium perchlorate (LiClO_4_, 99.9%, anhydrous), tetraglyme (TEGDME, 99.9%, anhydrous), and lithium iodide (LiI, anhydrous) were purchased from Sigma-Aldrich. Carbon black (Super P, >99%), poly(vinylidene fluoride) (PVDF), palladium(II) chloride (solution 20–25%, w/w), and melamine (C_3_H_6_N_6_, 99%) were purchased from Alfa Aesar. Lithium chips (16 × 0.25 mm^2^, 99.9%) were purchased from MTI Corporation.

### 2.2 Synthesis Methods

Graphene oxide (GO) was synthesized by an improved method as reported in our previous work.^24^ According to this method, the mixture of acids, H_2_SO_4_/H_3_PO_4_ (360:40 mL), was slowly added into a mixture of KMnO_4_ (18.0 g) and graphite flakes (3.0 g). Then, the reaction mixture was heated to 50 °C and stirred at 300 rpm for 12 h. The reaction mixture was cooled down to room temperature and poured into ice (400 mL) while adding H_2_O_2_ (3 mL) dropwise into the mixture. After this step, the mixture was filtered and washed. The initial washing step was performed with 30% HCl solution, and this step was repeated until the supernatant became transparent. Then, the washing process was continued with DI water and ethanol until a neutral pH value was obtained. All washing processes were performed by centrifugation at 6000 rpm for 30 min. The resulting solid was dispersed in DI water by ultrasonication at a concentration of 1.0 mg mL^–1^.

Silica (SiO_2_) nanoparticles were synthesized according to the well-known method described by Stöber et al.^25^ Initially, the synthesized SiO_2_ nanoparticles were dispersed in DI water by sonication, and then HCl, Pluronic F108 including methyl group (−CH_3_), and DMDMS were added to this dispersion. After mixing for 24 h at 400 rpm, the suspension was neutralized by adding the required amount of NH_4_OH.

Finally, to obtain GO/SiO_2_ nanostructures (Figure S1), SiO_2_ dispersion and GO suspension were mixed for 24 h and then centrifuged for 1 h at 6000 rpm to let the solid part precipitate. Final drying was provided at RT inside the vacuumed desiccator. GO/SiO_2_ nanostructures were converted into rGO/SiO_2_ nanostructures after heat treatment at 900 °C for 4 h under an Ar atmosphere. To achieve a porous rGO structure, the SiO_2_ nanoparticles with ∼30 nm diameter (Figure S2) were etched using NaOH solution. The final rGOs (Figure S3) with the ∼30 nm pore size, which is considered as an ideal pore size for the optimum oxygenation of the cathode structure,^26^ were obtained after one more heat treatment at 900 °C for 5 h under an Ar atmosphere.

PdCl_2_ (120 μL) was initially dissolved in a 4 mL aqueous solution containing 5 vol % HCl, and then by adding ethylene glycol (60 mL), the solution was stirred until accomplishing homogenization. Porous rGO (72 mg) was added to this solution to obtain the Pd-loaded rGO. After 30 min of sonication, the solution was transferred to the reflux system. Initially, the pH of the solution was adjusted to 12, and then the solution was heated to 130 °C to hold at this temperature for 2 h. After this step, the residue was filtrated and washed several times with DI water and ethanol. The final step was drying of the obtained porous Pd@rGO nanostructure (Figure S4) at 80 °C inside the vacuum desiccator.

To prepare g-C_3_N_4_/rGO nanocomposites, melamine and rGO were mixed in ethanol at 50 °C until all of the methanol evaporated. After complete drying, melamine and rGO were put in a crucible (30 mL) with a cover and heated up to 550 °C at a rate of 3 °C min^–1^ and then kept at this temperature for another 3 h under continuous Ar flow (1.2 L min^–1^) and subsequently cooled down to room temperature. The compositions of the nanocomposites are provided in Table S1, and the color change with increasing rGO content in the synthesized rGO/g-C_3_N_4_ nanocomposites is shown in Figure S5.

### 2.3 Electrode Preparations and Electrochemical Measurements

Photocurrent measurements were made by the linear sweep voltammetry technique in a conventional three-electrode cell with a platinum wire as the auxiliary electrode and a Ag/AgCl (saturated KCl) as the reference electrode on a Gamry Reference 3000 workstation. A solar simulator (A-type 150 W, 1–3 sun, Xenon lamp, AMO filters; 400–700 nm wavelength) was used as the light source. The working electrode was prepared on ITO glass (10 Ω cm^–2^) by loading a 0.05 mg cm^–2^ semiconductor using a spin coater. Nanocomposites were dissolved only in methanol before loading, and the working electrodes were dried at 100 °C for 12 h in a vacuum desiccator after loading. Measurements were conducted in a spectral cell containing 0.1 M KCl buffered by 0.1 M K_2_HPO_4_ to pH 7. Mott–Schottky measurements were conducted using the same set-up by loading 0.8 mg cm^–2^ semiconductor on ITO. Before loading, nanocomposites were dissolved in a methanol (3.2 mL)/NMP (6.8 mL) mixture including 0.2% PEG4000, and after loading, the working electrodes were dried at 100 °C for 12 h in a vacuum desiccator.

Photoassisted charging of Li-ion oxygen batteries was carried out using a homemade cell in an oxygen cabin, which had 1 bar positive oxygen pressure during the measurements, as shown in Figure S6. The cell was assembled in an Ar-filled glovebox with H_2_O and O_2_ levels less than 0.1 ppm. Lithium metal was used as both counter and reference electrodes, and the glass microfiber filter (Whatman) was used as a separator. Porous Pd@rGO/Super P carbon black/PVDF were mixed (80:10:10 wt %) in NMP, and the slurry was coated onto one side of 16 mm diameter GDL (TGP-H-060) with a loading rate of 0.1 mg cm^–2^ as a cathode (Figure S7). The semiconductor nanocomposite (dissolved only in methanol) was coated on the other side of GDL with a loading rate of 0.05 mg cm^–2^ as a photoelectrode (Figure S7). Before the battery assembly, the electrodes were dried in a vacuum oven at 100 °C overnight. The cathode side of GDL was facing the anode, and the photoelectrode side of GDL was facing the spectral glass window mounted on the homemade cell. LiClO_4_ (0.5 M), which is known for its remarkable performance in terms of the rechargeability of the Li-ion oxygen batteries,^27^ and 0.05 M LiI dissolved in TEGDME, which is known for its superior Li^+^ transport and good Li-salt solubility,^27^ was used as an electrolyte for the photoassisted charging. LiI was excluded from some charging tests conducted without illumination. The discharge and charge tests were performed galvanostatically, and the discharge cutoff potential was 2.0 V_Li^+^/Li_. The charge cutoff potentials were 3.6 and 4.2 V_Li^+^/Li_ for the photoassisted and dark charging, respectively. The current densities changed between 10 mA g^–1^ (10^–3^ mA cm^–2^) and 500 mA g^–1^ (5 × 10^–2^ mA cm^–2^).

### 2.4 Structural Characterizations

X-ray diffraction (XRD) analyses were performed on a PANalytical Empyrean diffractometer with Cu Kα radiation at a scanning rate of 2° min^–1^. The morphologies were examined with a ZEISS Ultraplus scanning electron microscope (SEM). FTIR measurements were conducted using a PerkinElmer Spectrum Two. UV/vis spectra were collected with a Cary 5000 UV/vis/NIR spectrometer with a diffuse reflectance accessory between 200 and 800 nm. The Raman analysis of the electrodes was conducted by Raman spectroscopy (Renishaw Raman inVia microscope) with an excitation laser of 633 nm.

## 3 Results and Discussion

### 3.1 Structure and Morphology

g-C_3_N_4_/rGO nanocomposites were synthesized in a large composition range (g-C_3_N_4_/1% rGO to g-C_3_N_4_/75% rGO) to determine the structural and morphological changes clearly (Table S1). The color change shown in Figure S5 shows that while the pure g-C_3_N_4_ is bright yellow, the color of the synthesized nanocomposites changes from slight gray for g-C_3_N_4_/1% rGO to gray for g-C_3_N_4_/3% rGO and dark gray for g-C_3_N_4_/25% rGO. As the rGO content increases further (g-C_3_N_4_/50% rGO), the color of the nanocomposite becomes more blackish by approaching that of pure rGO. Since the density of rGO is much lower than that of g-C_3_N_4_, the volume percentage of rGO increases enormously as its weight percentage increases, and the nanocomposite gathers the appearance of pure rGO even at low weight percentages of rGO.

**Figure 1:**
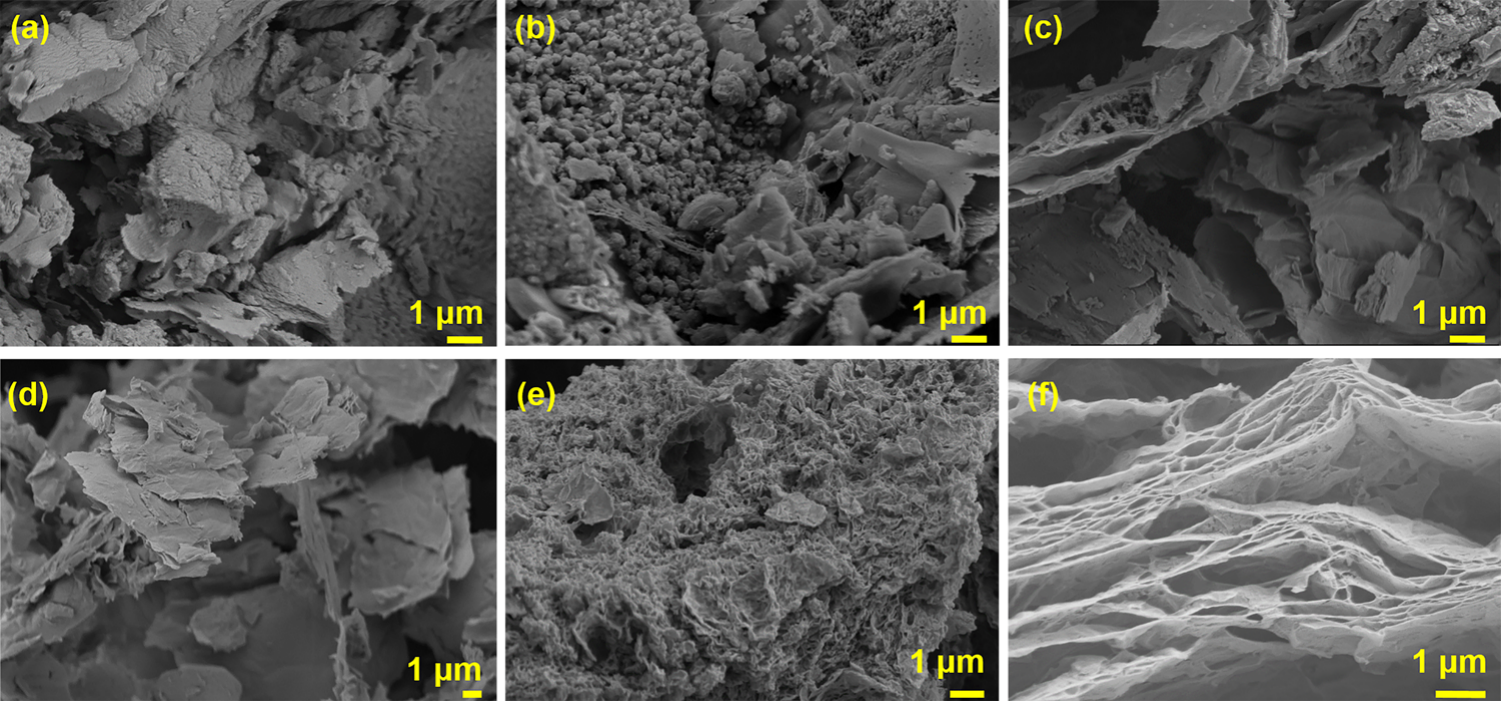
Morphologies of the synthesized (a) pure g-C_3_N_4_, (b) g-C_3_N_4_/3% rGO, (c) g-C_3_N_4_/10% rGO, (d) g-C_3_N_4_/25% rGO, (e) g-C_3_N_4_/50% rGO, and (f) pure rGO.

The morphologies of the synthesized nanocomposites are shown in Figure 1. Morphologies of the nanocomposites containing rGO up to 25% (Figure 1b–d) are very similar to that of pure g-C_3_N_4_ (Figure 1a), which has a slatelike stacked lamellar microstructure. Further increase in the rGO (Figure 1e) causes the nanocomposite morphology to become similar to that of rGO, which has a porous layered structure (Figure 1f).

**Figure 2:**
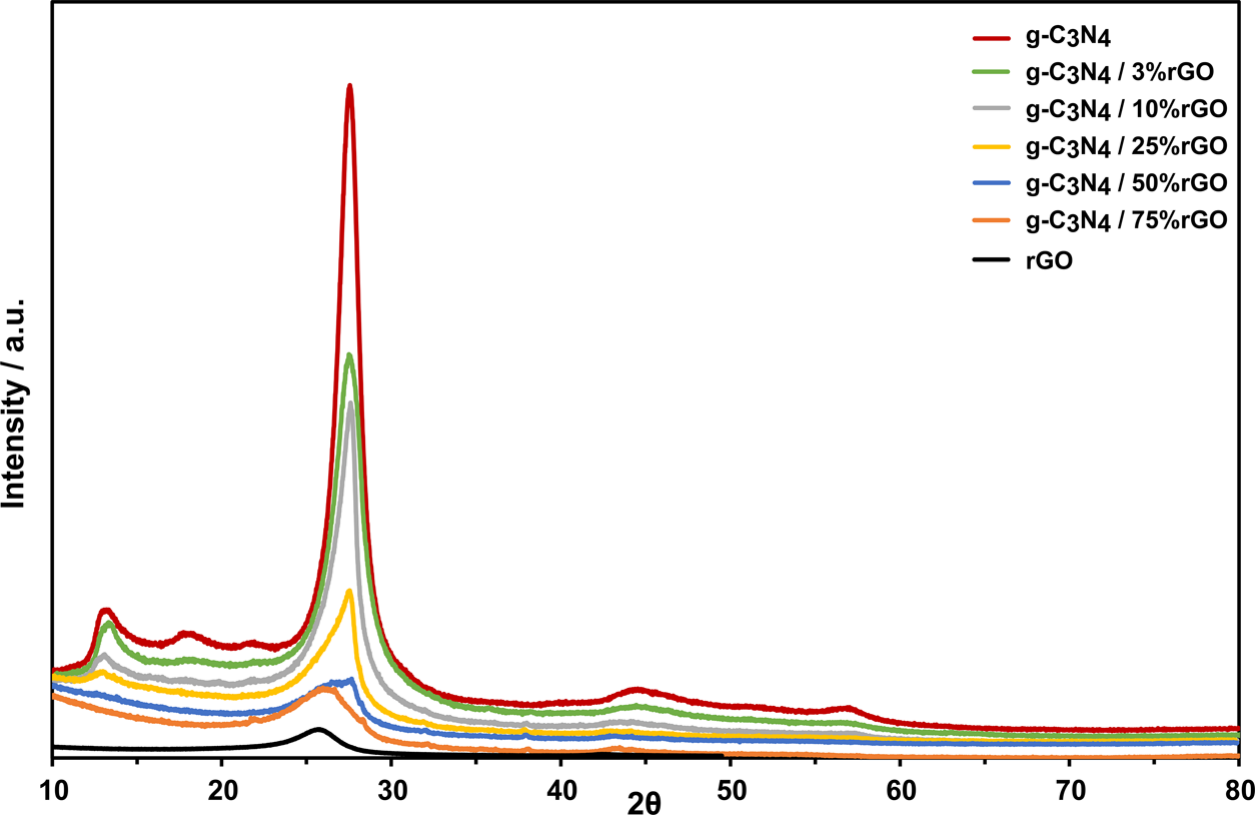
XRD patterns of pure g-C_3_N_4_, g-C_3_N_4_/3% rGO, g-C_3_N_4_/10% rGO, g-C_3_N_4_/25% rGO, g-C_3_N_4_/50% rGO, g-C_3_N_4_/75% rGO, and pure rGO.

Figure 2 shows the XRD patterns of the pure g-C_3_N_4_, rGO, and g-C_3_N_4_/rGO nanocomposites with different rGO ratios. The broad peak located at around 26° in the rGO pattern is ascribed to the presence of the loosely stacked sheets.^28^ A strong characteristic (002) peak at 27.6° in the pure g-C_3_N_4_ pattern is also accepted as the indication of the layered structure.^15^ Another characteristic peak (100) at around 13.2° in the pure g-C_3_N_4_ pattern in Figure 2 corresponds to the in-plane ordering of tri-s-triazine units.^29^ g-C_3_N_4_/rGO nanocomposites containing rGO up to 25% have almost the same characteristic peaks (almost the same 2θ position) as the pure g-C_3_N_4_ in Figure 2. g-C_3_N_4_/50% rGO, however, has two small peaks at around 27.55 and 26.1° and shows kind of a structural transition. g-C_3_N_4_/75% rGO has one peak at 26° and its pattern resembles that of pure rGO. The reported TEM images also support the multilayer structural characteristics of the g-C_3_N_4_/rGO nanocomposites.^14,15,19,20^

**Figure 3:**
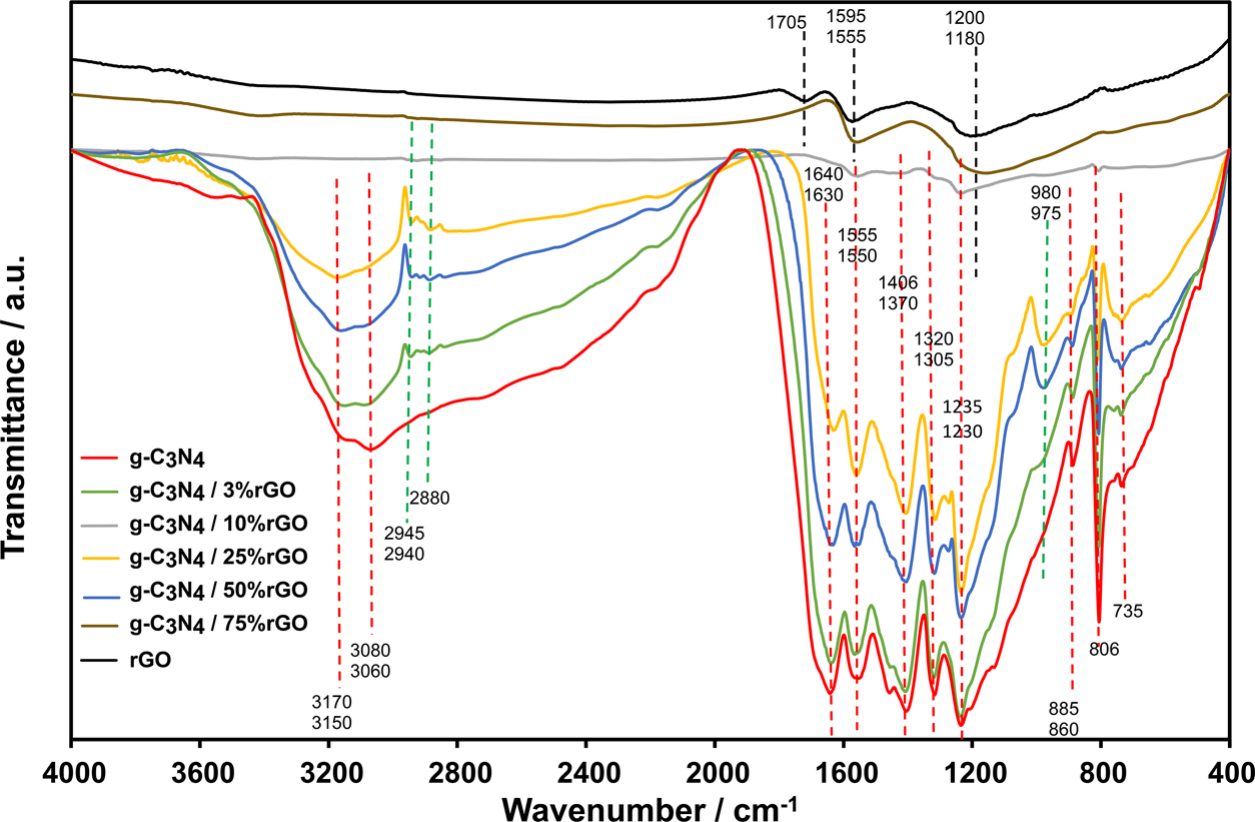
FTIR spectra of pure g-C_3_N_4_, g-C_3_N_4_/3% rGO, g-C_3_N_4_/10% rGO, g-C_3_N_4_/25% rGO, g-C_3_N_4_/50% rGO, g-C_3_N_4_/75% rGO, and pure rGO.

The structural changes in the synthesized g-C_3_N_4_/rGO nanocomposites can be identified more sensitively by FTIR spectra, as shown in Figure 3. To present clearly, peaks belonging to pure g-C_3_N_4_, pure rGO, and g-C_3_N_4_/rGO nanocomposites (novel ones) are shown by red, black, and green dashed lines, respectively, in Figure 3. The broad absorption peaks between 3000 and 3200 cm^–1^, belonging to pure g-C_3_N_4_, are associated with N=H stretching (due to residual amino groups or absorbed water).^30^ Spectrum peaks located between 1200 and 1650 cm^–1^ are ascribed to the typical stretching modes of CN heterocycles.^30^ Peaks located in the range change from 735 to 806 cm^–1^ and at 885 cm^–1^ are attributed to the triazine ring stretching and N–H band deformation modes, respectively.^30^ The spectrum peaks at 1705, 1595, and 1200 cm^–1^, belonging to pure rGO, are ascribed to the stretching vibrations of C=O, graphitic domains confirming the formation of sp^2^ carbon structure, and C–C/C–OH, respectively, as shown in Figure 3.^31^ While g-C_3_N_4_/rGO nanocomposites containing rGO of up to 25% have all of the characteristic peaks of the pure g-C_3_N_4_, g-C_3_N_4_/50% rGO and g-C_3_N_4_/75% rGO nanocomposites have all of the characteristic peaks of the pure rGO. Novel peaks (marked by green dashed lines) in the g-C_3_N_4_/rGO nanocomposites located in the range between 2880 and 2945 cm^–1^ (medium strength) and at 975 cm^–1^ (strong) can be attributed to the C–H stretching vibrations probably due to the interaction of rGO with the residual amino groups or absorbed water, and the C=C bond bending vibrations, respectively, in Figure 3. The strong peak that appeared belonging to the C=C bond evidences the bond formation between g-C_3_N_4_ and rGO during the decomposition of melamine to g-C_3_N_4_ at 550 °C.

### 3.2 Optical Properties

**Figure 4:**
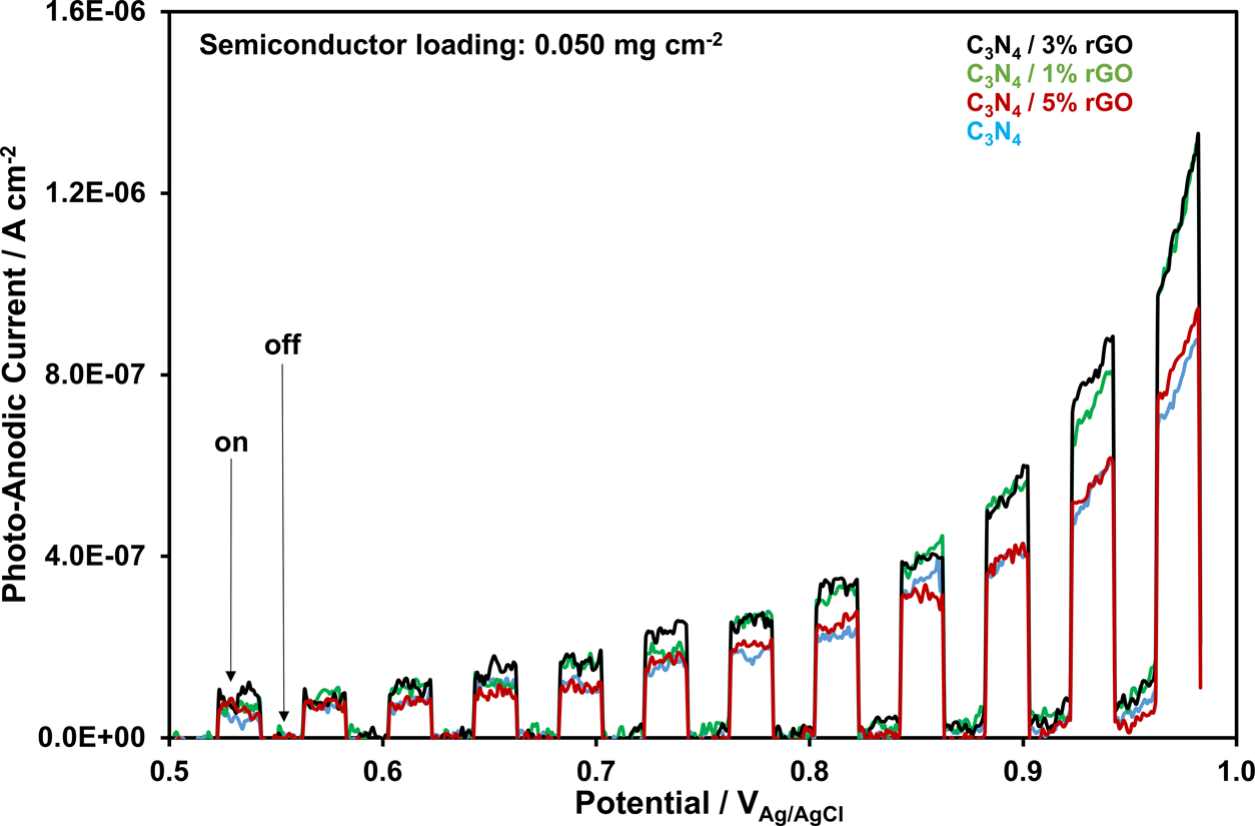
Photoanodic currents of pure g-C_3_N_4_, g-C_3_N_4_/1% rGO, g-C_3_N_4_/3% rGO, and g-C_3_N_4_/5% rGO nanocomposites.

The photoanodic currents, via on/off cycles under the visible-light irradiation, were determined for all of the synthesized nanocomposites (Table S1) by the linear sweep voltammetry techniques, although only those of g-C_3_N_4_/1% rGO, g-C_3_N_4_/3% rGO, and g-C_3_N_4_/5% rGO are shown in Figure 4. The photoanodic currents of pure g-C_3_N_4_ are also provided in Figure 4 for comparison. The incorporation of 1% rGO results in about a 40% increase in the photocurrents, especially at high anodic potentials. The increase in the rGO content up to 3% causes slightly more improvement in the photocatalytic efficiency of the nanocomposite. The enhancement in the photocurrents with the presence of rGO implies more efficient visible light utilization. The further increase in the rGO content (g-C_3_N_4_/5% rGO), however, degrades the visible light utilization, and the photocurrents show a decrease since more incident light is absorbed by rGO instead of g-C_3_N_4_,^15^ as shown in Figure 4. The further increase in the rGO content results in higher degradation in the photocurrents, and the photocurrent data belonging to the nanocomposites with higher rGO content are excluded in Figure 4.

**Figure 5:**
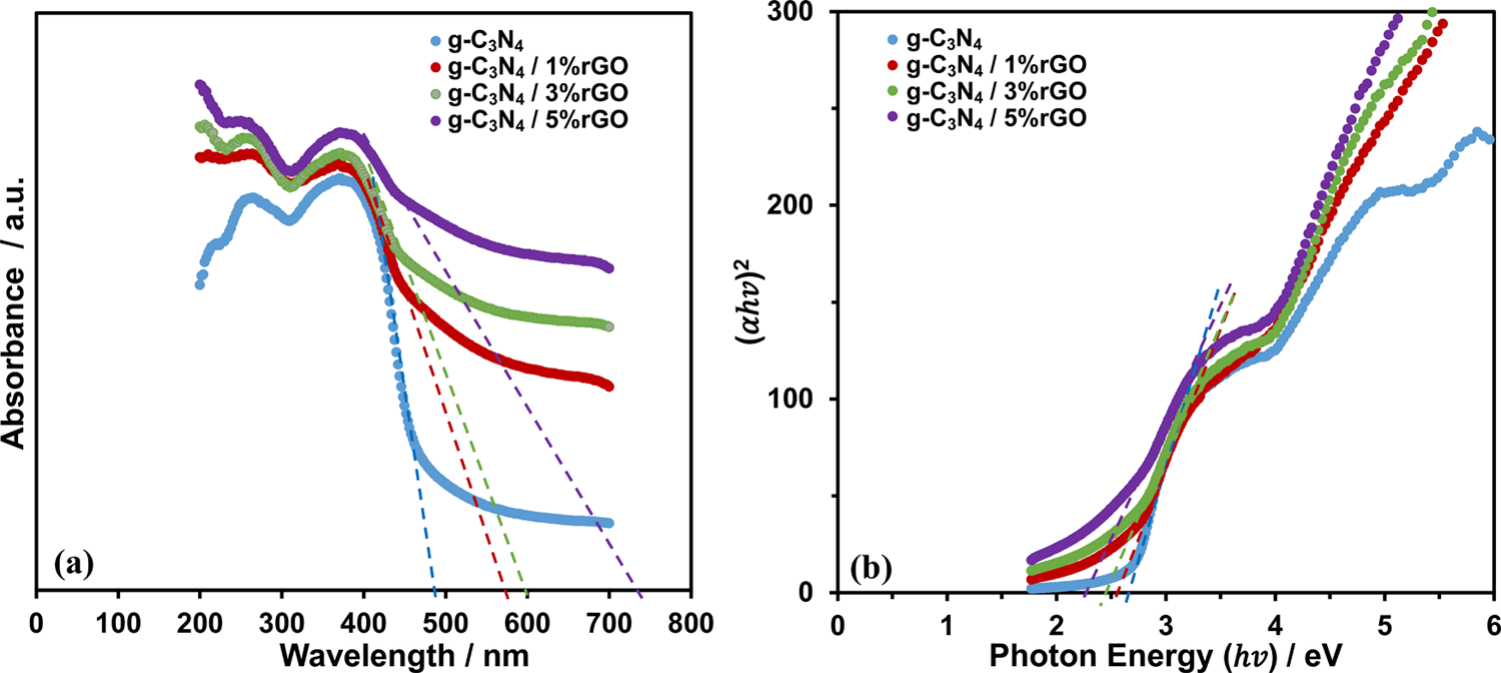
(a) Absorption edges and (b) optical band gaps of g-C_3_N_4_, g-C_3_N_4_/1% rGO, g-C_3_N_4_/3% rGO, and g-C_3_N_4_/5% rGO.

The UV–vis diffuse reflectance spectra of the pure g-C_3_N_4_ and nanocomposites are provided in Figure 5a. The absorption band edge of g-C_3_N_4_ is 485 nm, and it increases to 565, 595, and 730 nm for g-C_3_N_4_, g-C_3_N_4_/1% rGO, g-C_3_N_4_/3% rGO, and g-C_3_N_4_/5% rGO, respectively. The optical band gaps (*E*_g_’s) can be obtained by the Tauc plot,^32^ as shown in Figure 5b; they are 2.7, 2.55, 2.45, and 2.25 eV for g-C_3_N_4_, g-C_3_N_4_/1% rGO, g-C_3_N_4_/3% rGO, and g-C_3_N_4_/5% rGO, respectively. The shift in the absorption band edges to the higher wavelengths and the corresponding narrowing in the optical band gaps as the rGO content of the nanocomposites increases are attributed to a red shift in the absorption band edge.^15^ The observation of a red shift in g-C_3_N_4_/rGO nanocomposites is believed to be associated with the formation of a novel chemical bonding during the synthesis of the nanocomposites^15^ as it is also pointed out in this work in Figure 3.

**Figure 6:**
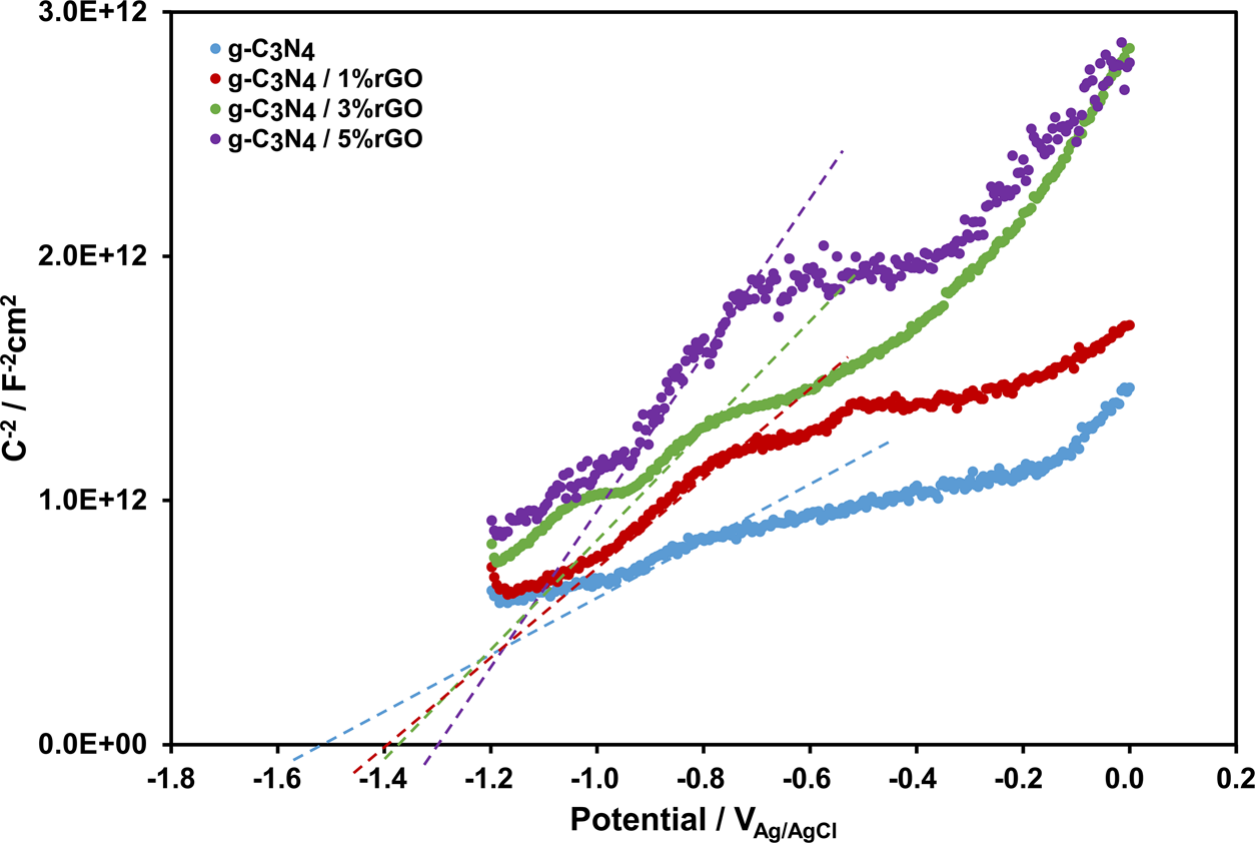
Mott–Schottky plots of g-C_3_N_4_, g-C_3_N_4_/1% rGO, g-C_3_N_4_/3% rGO, and g-C_3_N_4_/5% rGO obtained at 2000 Hz.

The photooxidation characteristics of the photocatalysts are determined by the valance band (VB) edge potential, which can be calculated if the optical band gap (*E*_g_) and the conduction band (CB) edge potentials are obtained by simply adding them up. If the flat band potential is assumed approximately equal to the CB edge potential, Mott–Schottky plots, as in Figure 6, can be utilized in the determination of the CB edge potentials; they are −1.56, −1.42, −1.39, and −1.31 V_Ag/AgCl_ for g-C_3_N_4_, g-C_3_N_4_/1% rGO, g-C_3_N_4_/3% rGO, and g-C_3_N_4_/5% rGO, respectively. *E*_g_ values, CB, and calculated VB edge potentials are all tabulated in Table 1.

**Table 1:** *E*_g_, CB, and VB Values of g-C_3_N_4_, g-C_3_N_4_/1% rGO, g-C_3_N_4_/3% rGO, and g-C_3_N_4_/5% rGO

	CB edge potential			
semiconductor	V_Ag/AgCl_	V_Li^+^/Li_	optical band gap (eV)	VB edge potential (V_Li^+^/Li_)
g-C_3_N_4_	–1.56	1.70	2.70	4.40
g-C_3_N_4_/1% rGO	–1.42	1.84	2.55	4.39
g-C_3_N_4_/3% rGO	–1.39	1.87	2.45	4.32
g-C_3_N_4_/5% rGO	–1.31	1.95	2.25	4.20

### 3.3 Photoassisted Charging of Li-Ion Oxygen Batteries

**Figure 7:**
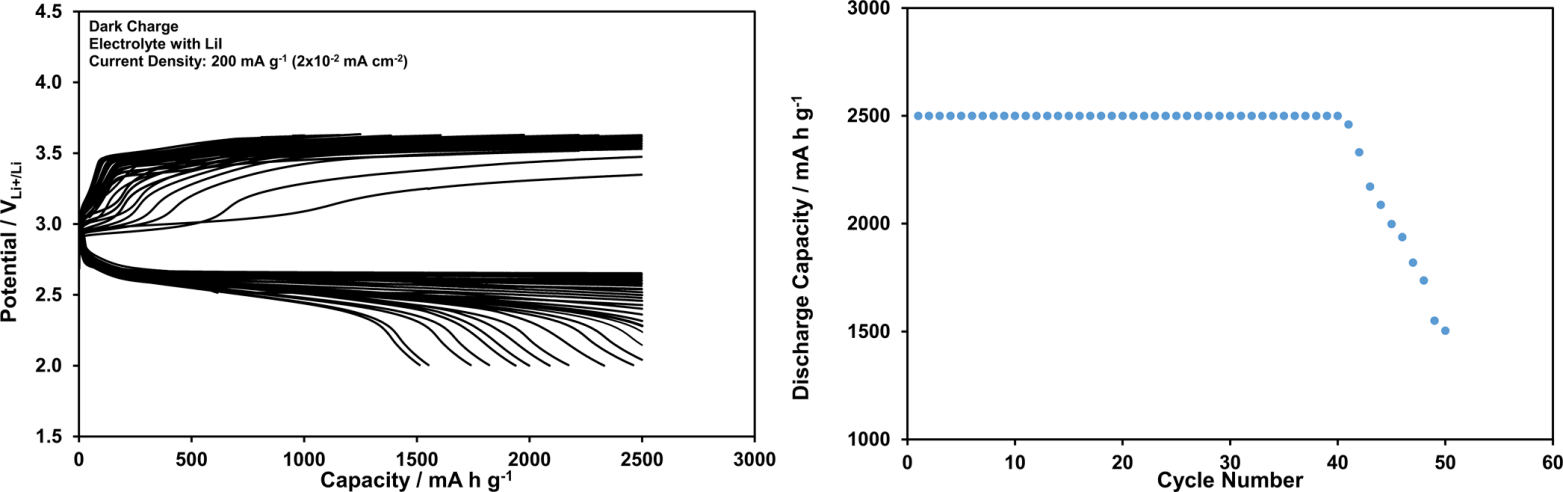
Discharge and charge curves recorded at 2500 mAh g^–1^ (0.25 mAh cm^–2^) constant capacity and 200 mA g^–1^ (2 × 10^–2^ mA cm^–2^) current density for the Li-ion oxygen battery with electrolyte including LiI under the dark conditions, and dependency of the discharge capacity on the cycle number.

The discharge and charge processes in the Li-ion oxygen batteries are controlled mainly by the kinetics of reaction 1
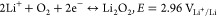
1The insulating characteristics of Li_2_O_2_ result in extended oxidation (decomposition) overpotential during the charging, and the charge potential increases up to levels well above 2.96 V_Li^+^/Li_, as illustrated in Figure S8, for the Li-ion oxygen battery, which includes the Pd@porous rGO (Figure S4) cathode, at a constant capacity of 2500 mAh g^–1^ (0.25 mAh cm^–2^) for 50 cycles. The use of the I^–^/I_3_^–^ couple as the redox shuttle between the porous cathode surface and Li_2_O_2_ particles helps to reduce the charging potential down to the redox potential of reaction 2, as shown in Figure 7. Obviously, when the data in Figures S8 and 7 are compared, there is no considerable improvement in the battery performance with the redox shuttling in the absence of photoassisted charging since the charge potential approaches to the redox potential (acts as cutoff potential) of reaction 2 after a few discharge–charge cycles.
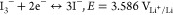
2Under the illumination during the charging process (photoassistance), I^–^ ions are oxidized to I_3_^–^ ions by the photoexcited holes of the photocatalyst if the VB edge potential of the photocatalyst is greater than the redox potential of reaction 2.^5,7^ Subsequently, I_3_^–^ ions diffuse to the cathode surface and spontaneously oxidize Li_2_O_2_ to Li^+^ ions and O_2_, while they reduce back to I^–^ ions to complete the cycle since the redox potential of reaction 2 is greater than that of reaction 1. Meanwhile, the photoexcited electrons of the photocatalyst flow to the anode to reduce Li^+^ ions, and the charge potential of the Li-ion oxygen battery is compensated by the generated photovoltage,^5,7^ if only the CB edge potential of the photocatalyst is lower than the redox potential of reaction 1. The rule is that the lower the CB edge potential, the higher the compensation of the charge potential by the photovoltage. The tuning ranges are provided by a diagram in Figure S9 for a clear presentation.

**Figure 8:**
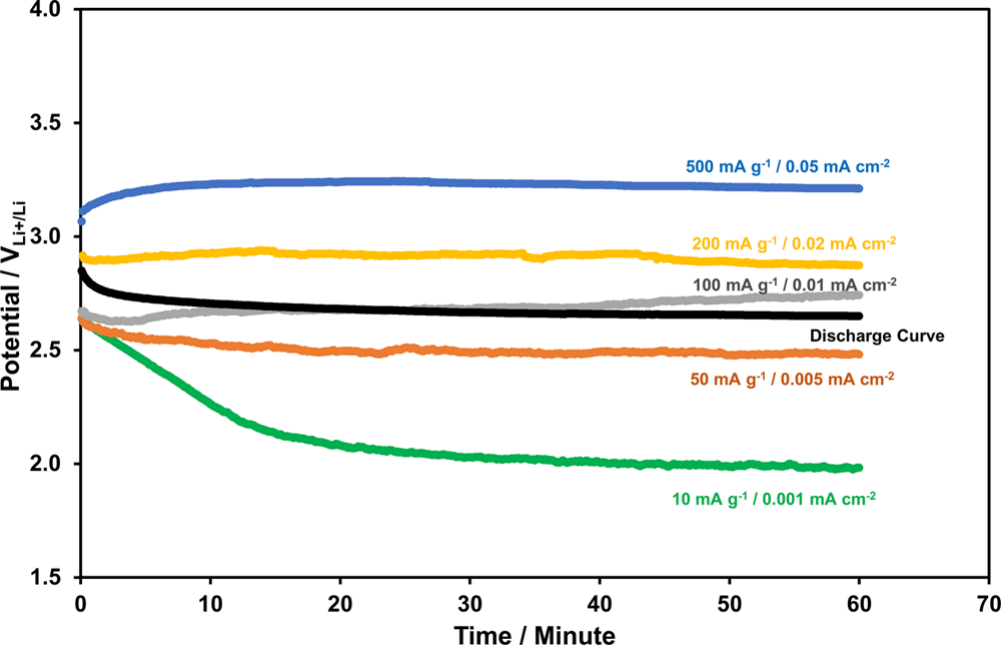
1 h-long charge curves, at various current densities, of the Li-ion oxygen battery with the g-C_3_N_4_ photocatalyst and porous Pd@rGO cathode under the photoassisted charging conditions. The discharge curves, for the comparison, are also provided.

**Figure 9:**
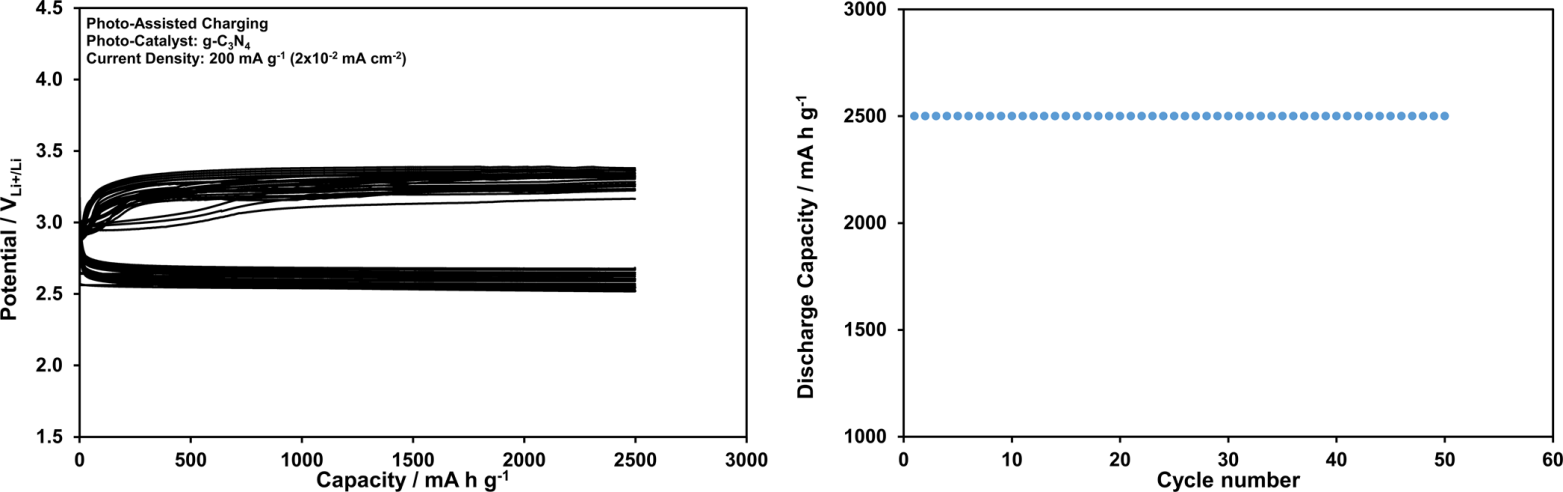
Discharge and charge curves recorded at 2500 mAh g^–1^ (0.25 mAh cm^–2^) constant capacity and 200 mA g^–1^ (2 × 10^–2^ mA cm^–2^) current density for the Li-ion oxygen battery with the g-C_3_N_4_ photocatalyst, porous Pd@rGO cathode, and electrolyte including LiI under the photoassisted conditions, and dependency of the discharge capacity on the cycle number.

The 1 h-long charge curves (at various current densities) of Li-ion oxygen batteries with the g-C_3_N_4_ photocatalyst, porous Pd@rGO cathode, and electrolyte including LiI under the photoassisted charging condition are shown in Figure 8. The discharge curve is also provided for comparison in Figure 8. The charge potential decreases down to 2 V_Li^+^/Li_ at 10 mA g^–1^ (0.001 mA cm^–2^), and it remains below the discharge potential (2.65 V_Li^+^/Li_) at 50 mA g^–1^ (0.005 mA cm^–2^). The charge potential remains at around the discharge potential level at 100 mA g^–1^ (0.01 mA cm^–2^), and it increases to 2.9 and 3.2 V_Li^+^/Li_ at 200 mA g^–1^ (0.02 mA cm^–2^) and 500 mA g^–1^ (0.05 mA cm^–2^), respectively. To reveal the effect of photoassistance on the battery performance, the tests presented in Figure 7 are repeated under illumination, and the results are given in Figure 9. Obviously, the charge potentials remain under 3.5 V_Li^+^/Li_ at 200 mA g^–1^ (0.02 mA cm^–2^) with the photoassistance for 50 cycles and the Li-ion oxygen battery performance is improved significantly. This improvement is also illustrated in Figure S10 by comparing the total (charge + discharge) overpotentials collected from Figures 7 and 9 for 50 cycles.

**Figure 10:**
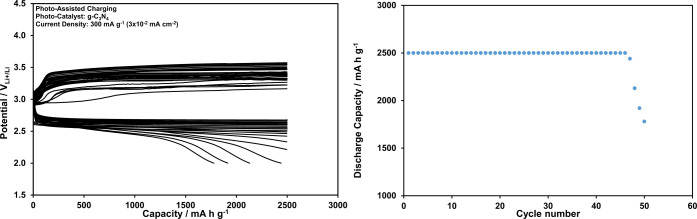
Discharge and charge curves recorded at 2500 mAh g^–1^ (0.25 mAh cm^–2^) constant capacity and 300 mA g^–1^ (3 × 10^–2^ mA cm^–2^) current density for the Li-ion oxygen battery with the g-C_3_N_4_ photocatalyst, porous Pd@rGO cathode, and electrolyte including LiI under the photoassisted conditions, and dependency of the discharge capacity on the cycle number.

Upon increasing the current density up to 300 mA g^–1^ (0.03 mA cm^–2^), however, as shown in Figure 10, the performance of the Li-ion oxygen battery degrades despite the presence of photoassistance. Further improvement in the battery performance under the illuminated conditions can be succeeded only by improving the photocatalyst performance.

**Figure 11:**
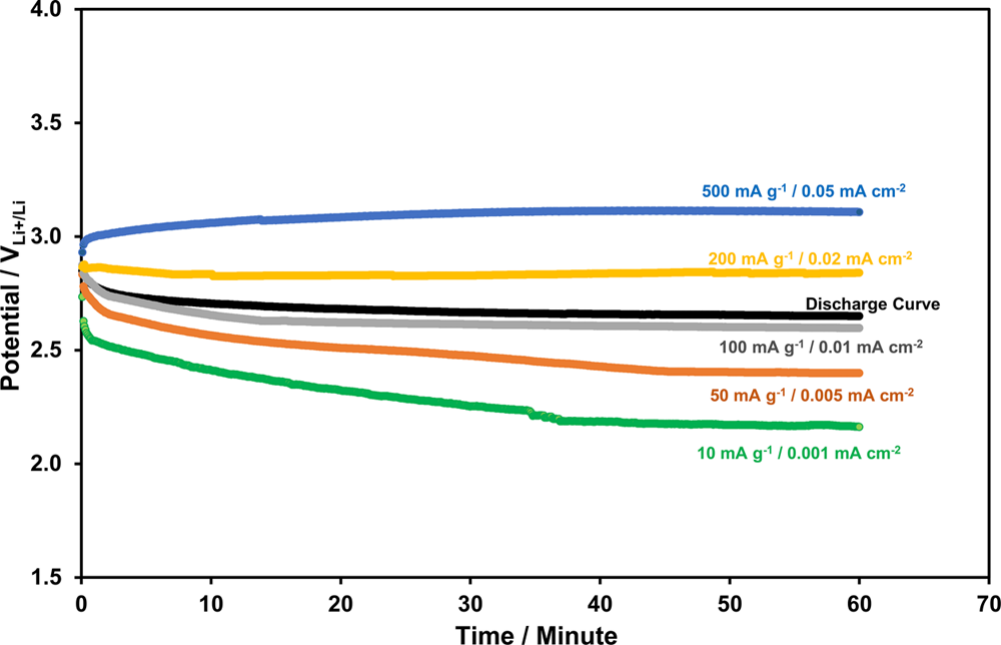
1 h-long charge curves, at various current densities, of the Li-ion oxygen battery with the g-C_3_N_4_/3% rGO photocatalyst and porous Pd@rGO cathode under the photoassisted charging conditions. The discharge curves, for the comparison, are also provided.

When the photocatalyst is replaced by g-C_3_N_4_/3% rGO, in the Li-ion oxygen battery, the photoassisted charging curves at the same current densities in Figure 8 become as in Figure 11. The charge potential at the lowest current density (0.001 mA cm^–2^) is 2.2 V_Li^+^/Li_, that is, it is slightly higher than the corresponding potential value present in Figure 8 (2 V_Li^+^/Li_) probably due to the increase in the CB edge potential (from 1.7 to 1.87 V_Li^+^/Li_) in Table 1. Upon increasing the current density, as shown in Figure 11, however, a slower increase in the charge potentials is observed as compared to the corresponding trend shown in Figure 8. For example, the charge potentials at 200 mA g^–1^ (0.02 mA cm^–2^) and 500 mA g^–1^ (0.05 mA cm^–2^) current densities are 2.8 and 3.1 V_Li^+^/Li_, respectively, as shown in Figure 11. Obviously, the increase in the conductivity and the light absorbance efficiency of the photocatalyst results in better charge potential compensation by the generated photovoltage of the photoelectrode, especially at high current densities.

**Figure 12:**
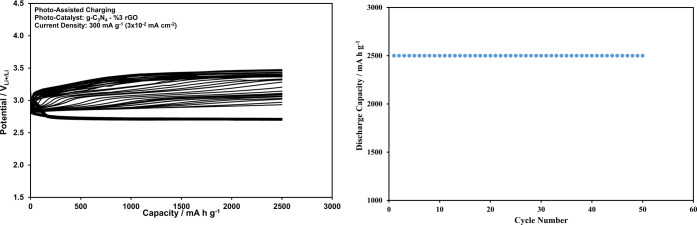
Discharge and charge curves recorded at 2500 mAh g^–1^ (0.25 mAh cm^–2^) constant capacity and 300 mA g^–1^ (3 × 10^–2^ mA cm^–2^) current density for the Li-ion oxygen battery with the g-C_3_N_4_/3% rGO photocatalyst, porous Pd@rGO cathode, and electrolyte including LiI under the photoassisted conditions, and dependency of the discharge capacity on the cycle number.

Replacement of the g-C_3_N_4_/3% rGO nanocomposite with the pure g-C_3_N_4_ as the photocatalyst in the Li-ion oxygen battery improves the cyclic performance of the battery at 300 mA g^–1^ (0.03 mA cm^–2^), as shown in Figure 12. If the data in Figures 10 and 12 are compared, the charge potentials remain lower than the redox potential of reaction 2, and the shift in the discharge potentials appears narrower in Figure 12. The comparison of the total overpotentials collected from Figures 10 and 12 for 50 cycles is also provided in Figure S11.

The SEM micrograph of the as-prepared cathode is provided in Figure S12a. Similar micrographs are shown in Figure S12b–d for the cathodes at the discharged state, the dark charged state, and the photoassisted charged state, respectively, to evaluate the post-test products after 50-cycle constant-capacity tests. The aggregate particles at the discharged state prove the formation of Li_2_O_2_ in the form of small platelet deposits (Figure S12b) instead of a toroid-like morphology, which highly depends on the applied current density and H_2_O concentration.^33,34^ Li_2_O_2_ particles decompose significantly in the charged states (Figure S12c,d). Li_2_O_2_ deposition/decomposition behavior is also observed by Raman spectra, as shown in Figure S13. Peroxide type O–O bonding in Li_2_O_2_ is typically evidenced by the strong Raman peak at ∼790 cm^–1^.^35^ This characteristic peak of Li_2_O_2_ is clearly visible at the discharged state. The intensity of this peak decreases in the dark charged state and significantly reduces in the photoassisted charged state, and the effectiveness of the photoassisted charging is evidenced clearly. The SEM micrographs of the photoelectrodes at the as-prepared condition and after the 50-cycle constant-capacity test are presented in Figure S14a,b, respectively. Micrographs indicate no detectable change in the morphology of the photoelectrode. Obviously, the nanocomposite photocatalyst can retain its stability during the long cyclic tests.

The overall results in this work indicate that the performance of the synthesized g-C_3_N_4_/rGO nanocomposites with the optimized content to have the improved photocatalyst efficiency is very encouraging to conduct further research on the photoassisted charging of the Li-ion oxygen batteries.

## 4 Conclusions

In conclusion, g-C_3_N_4_/rGO nanocomposites were synthesized, aiming at the effective photoassisted charging of the Li-ion oxygen battery using them as the photocatalysts. Optical characterizations showed the presence of red shifting in the optical absorption band edges of the nanocomposites. The reduction in the optical band gaps of the nanocomposites as a result of the red shift was ascribed to the formation of the novel C=C bonds between g-C_3_N_4_ and rGO during the synthesis. The g-C_3_N_4_/3% rGO nanocomposite was determined as the more efficient photocatalyst in the harvesting of visible light. The usage of this nanocomposite as the photoelectrode in the Li-ion oxygen battery resulted in a considerable reduction in the charge potential, especially at the high current densities and improved the battery cyclic performance. This work clearly indicated that following the efforts of Yu et al.,^5^ the photoassisted charging with the effective photocatalyst may open an important pathway for the researchers to conduct thorough studies to approach the final goal of making Li-ion oxygen batteries commercial by utilizing the sun, which is huge renewal energy source.

## Supporting Information Available

SEM images of the electrodes, Raman spectra, picture of the experimental set-up, overpotential curves, and the discharge–charge curves of the Li-ion oxygen battery under dark conditions (PDF)
