# Supplementary material for: Photoassisted Charging of Li-Ion Oxygen Batteries Using g-C3N4/rGO Nanocomposite Photocatalysts
Source: ACS Appl Mater Interfaces. 2022 Jul 21;14(30):34583–92. doi: 10.1021/acsami.2c05607 (PMC9354003; doi:10.1021/acsami.2c05607)
Supplement: Supplementary file 1 — am2c05607_si_001.pdf [file am2c05607_si_001.pdf]

Supporting Information

Photoassisted Charging of Li-Ion Oxygen Batteries Using g- C_3N_4/rGO Nanocomposite Photocatalysts

Ersu Lökçü, Nilay Kaçar, Meltem Çayirli, Reşat Can Özden and Mustafa Anik*

Department of Metallurgical and Materials Engineering, Eskisehir Osmangazi University, 26040, Eskisehir, Turkey

*Corresponding author: manik@ogu.edu.tr

ORCID IDs:

Ersu Lökçü, https://orcid.org/0000-0002-1972-627X
Nilay Kaçar, https://orcid.org/0000-0002-8297-6132
Meltem Çayirli, https://orcid.org/0000-0002-2006-6336
Reşat Can Özden, https://orcid.org/0000-0002-9242-3136
*Mustafa Anik, https://orcid.org/0000-0003-0166-7862

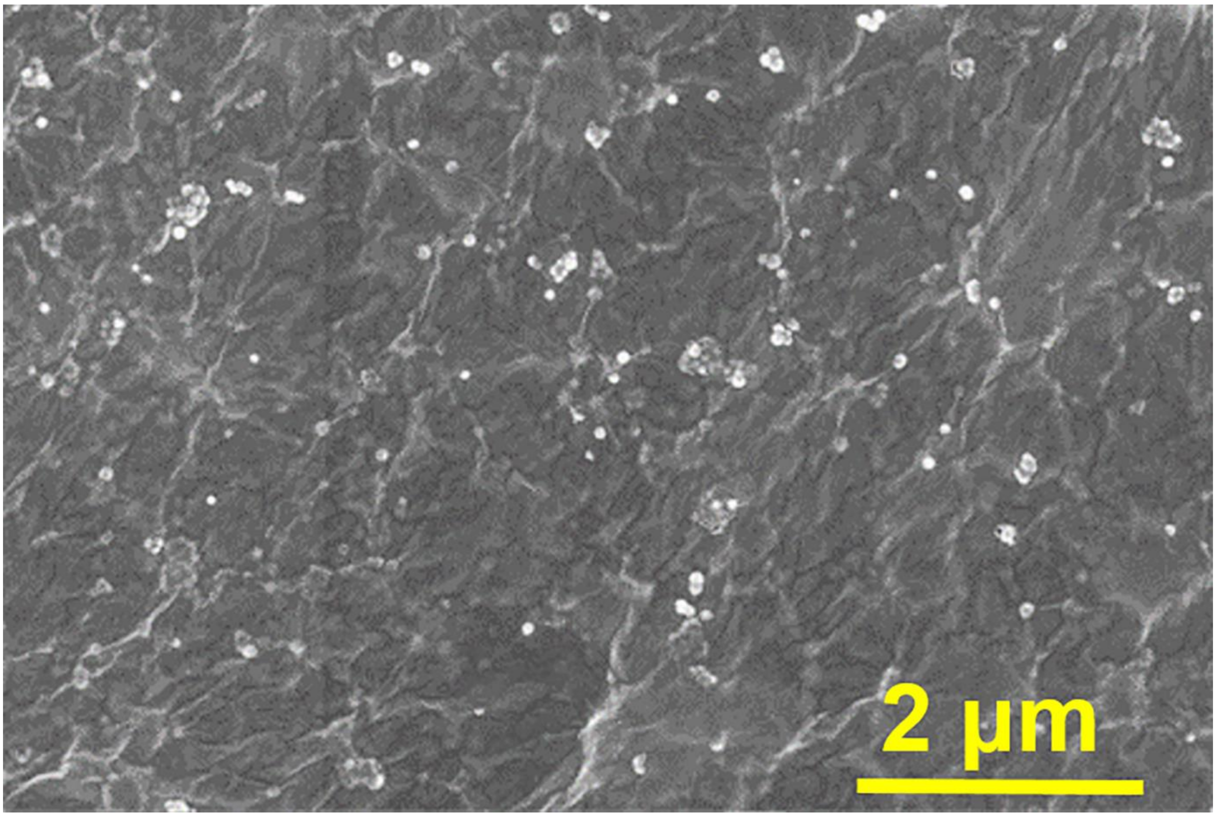

Figure S1. The morphology of the GO/SiO_2 nanostructure.

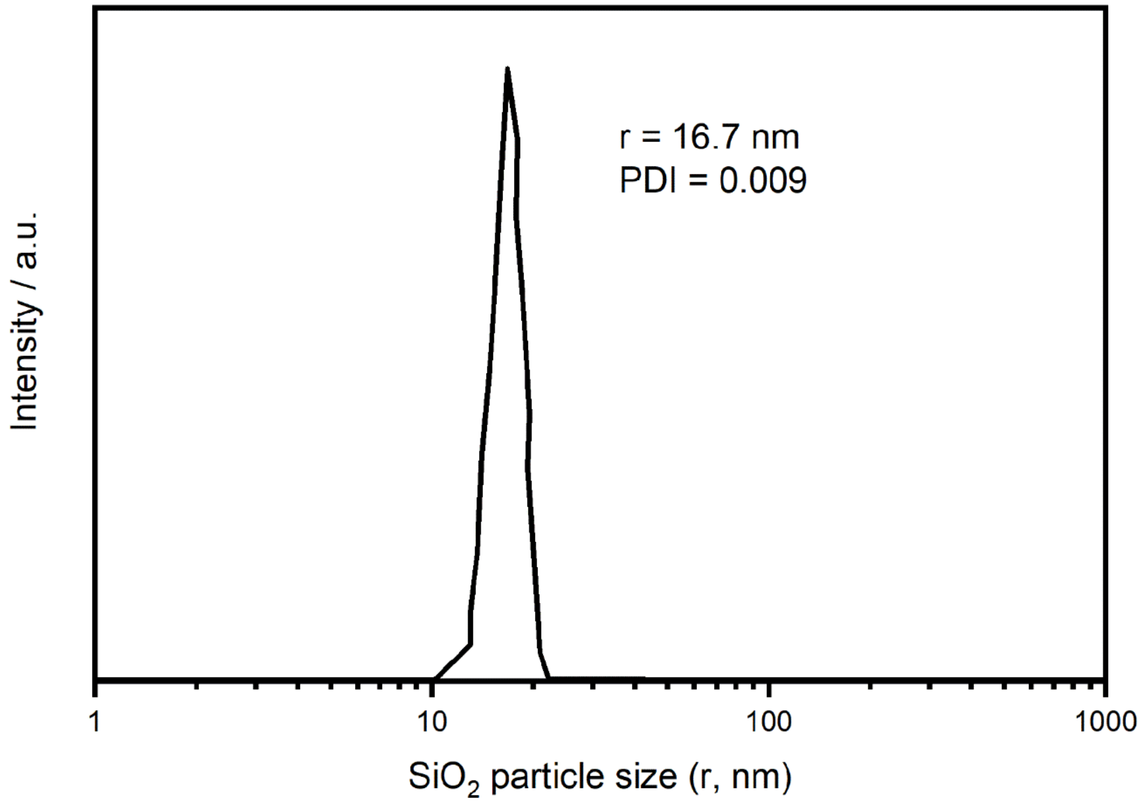

Figure S2. DLS analysis of the synthesized SiO_2 nanoparticles.

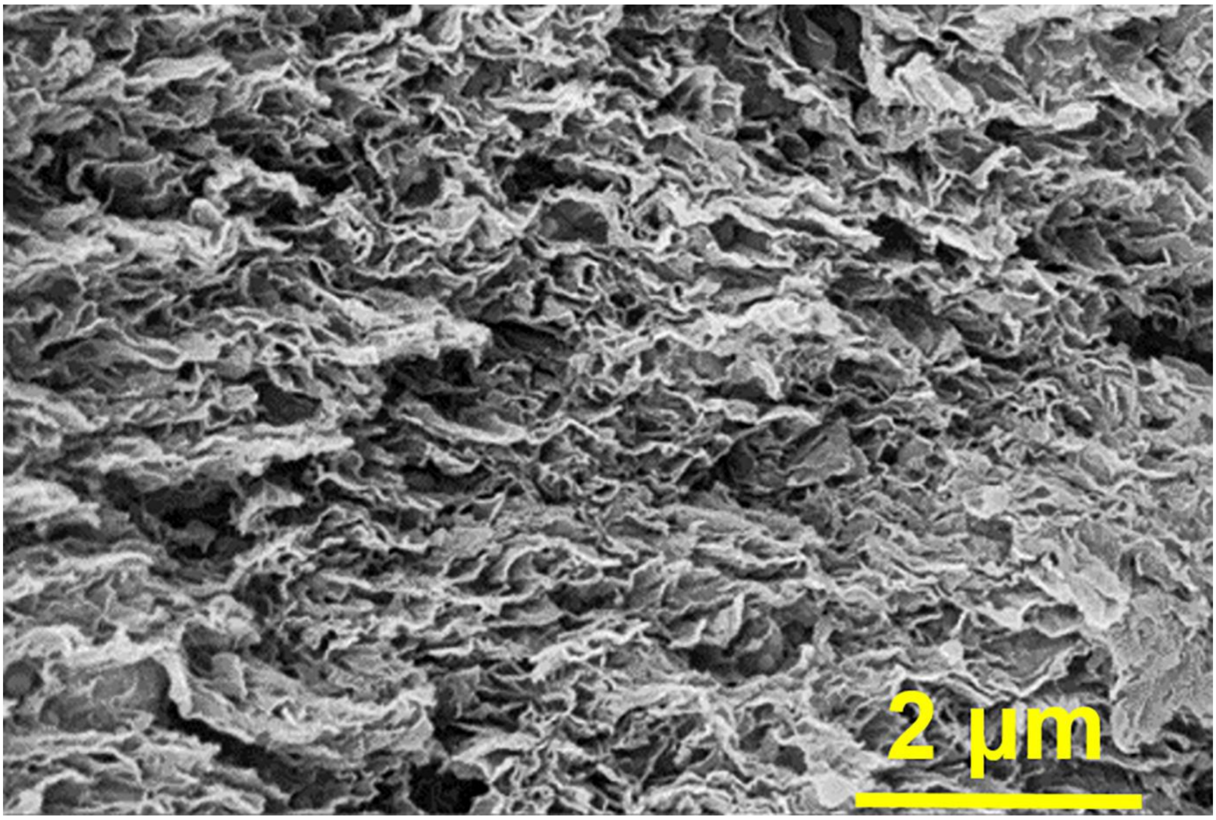

Figure S3. The morphology of the thermally reduced porous rGO. (Specific surface area is 440.6 $m^2\ g^{-1}$ and the pore volume is 2.15 $cm^3\ g^{-1}$).

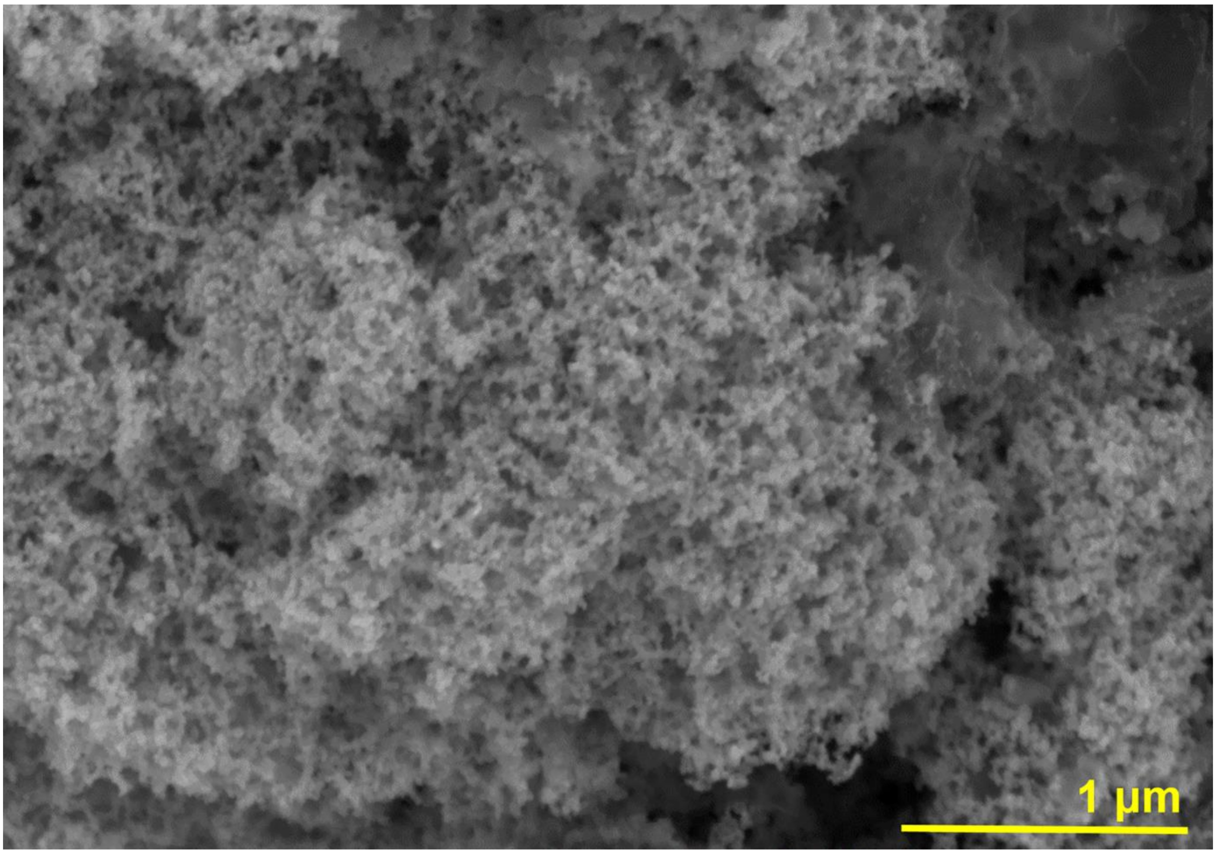

Figure S4. The morphology of the Palladium loaded porous rGO.

Table S1. The starting amounts of the constituents used in the synthesis of the nanocomposites and the obtained final amounts of the nanocomposites.

	Melamine (mg)	Porous rGO (mg)	rGO/g-C_3N_4 (mg)
1% rGO	1000	5	500 ± 1
3% rGO	650	10	335 ± 1
5% rGO	380	10	200 ± 1
10% rGO	380	20	195 ± 1
25% rGO	300	50	190 ± 1
50% rGO	200	90	185 ± 1
75% rGO	120	140	188 ± 1

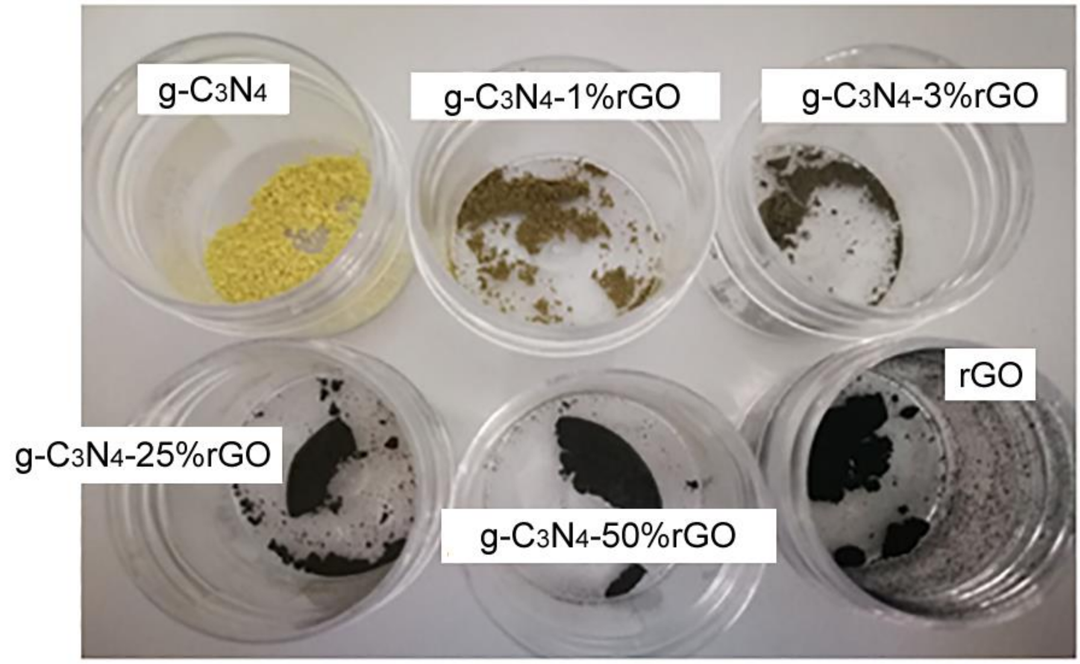

Figure S5. The color change in the synthesized nanocomposites as the rGO content increases.

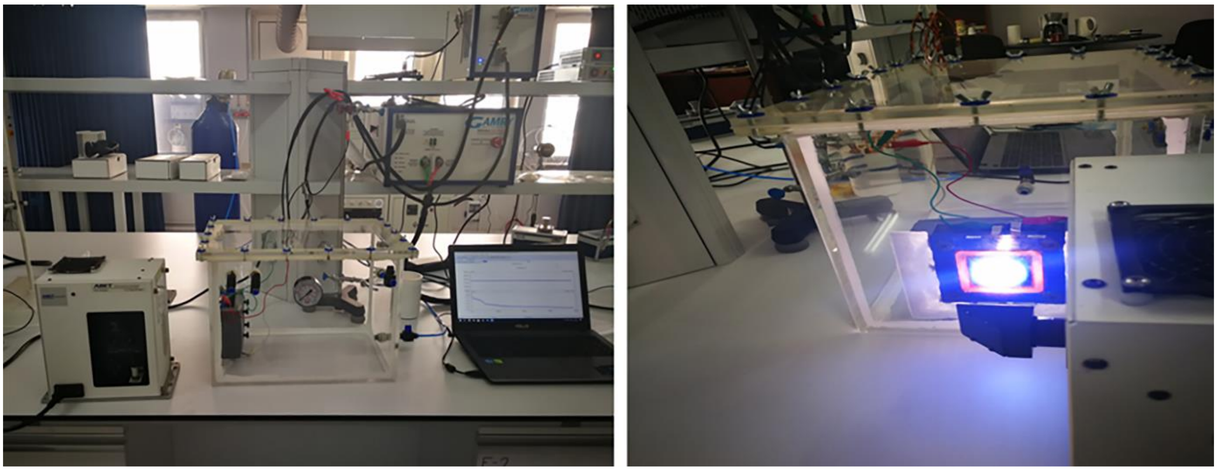

Figure S6. The pictures of the set-up, which is used in the photo-assisted charging, include the homemade oxygen cabin and cell.

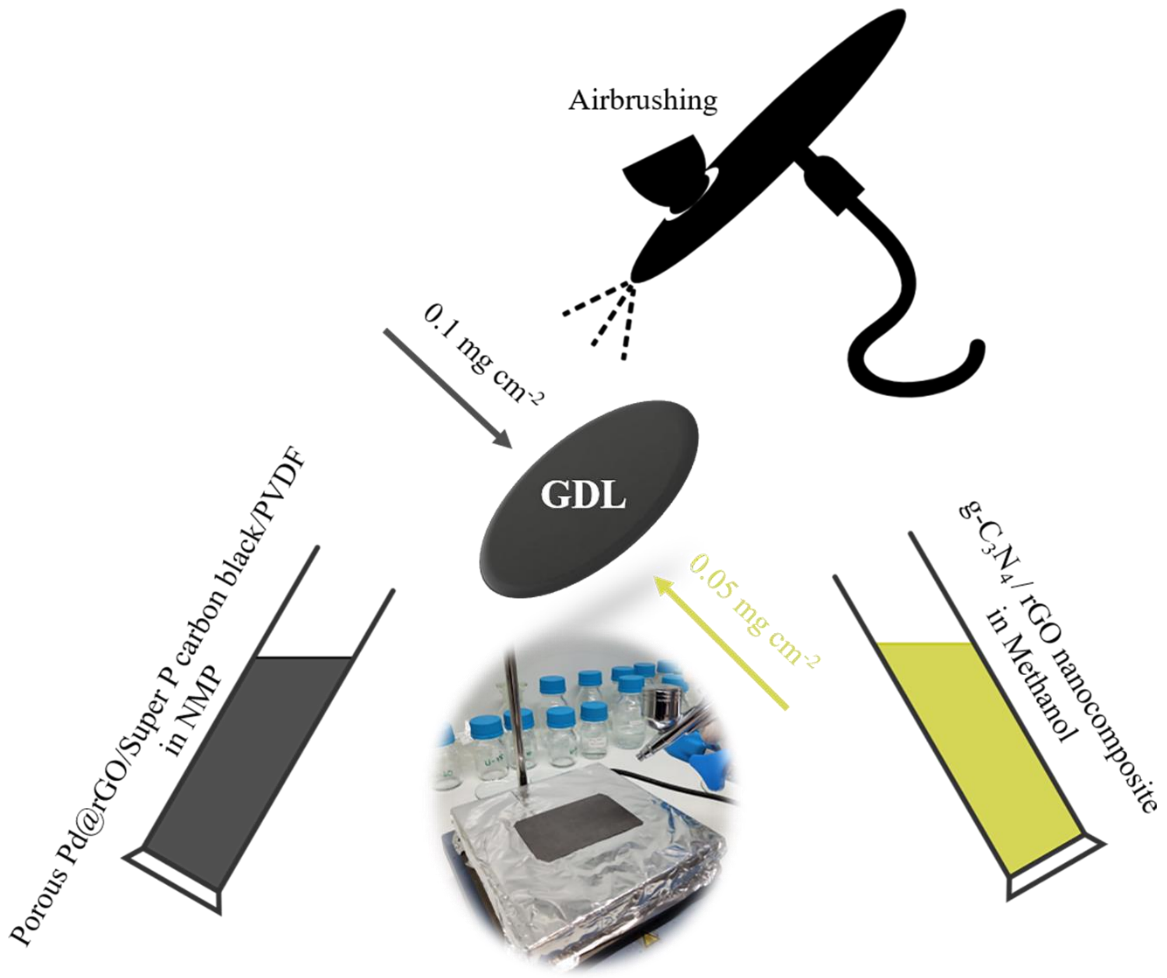

Figure S7. The optical image and schematic representation of the electrode preparation.

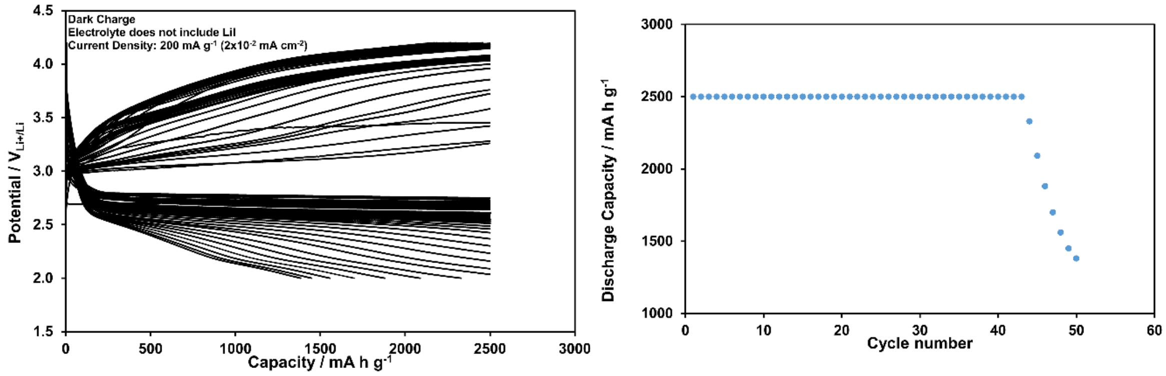

Figure S8. Discharge and charge curves gathered at 2500 mA h g^{-1} (0.25 mA h cm^{-2}) constant capacity and 200 mA g^{-1} (2x10^{-2} mA cm^{-2}) current density for Li-ion oxygen battery with electrolyte includes no LiI under the dark conditions, and dependency of the discharge capacity on the cycle number.

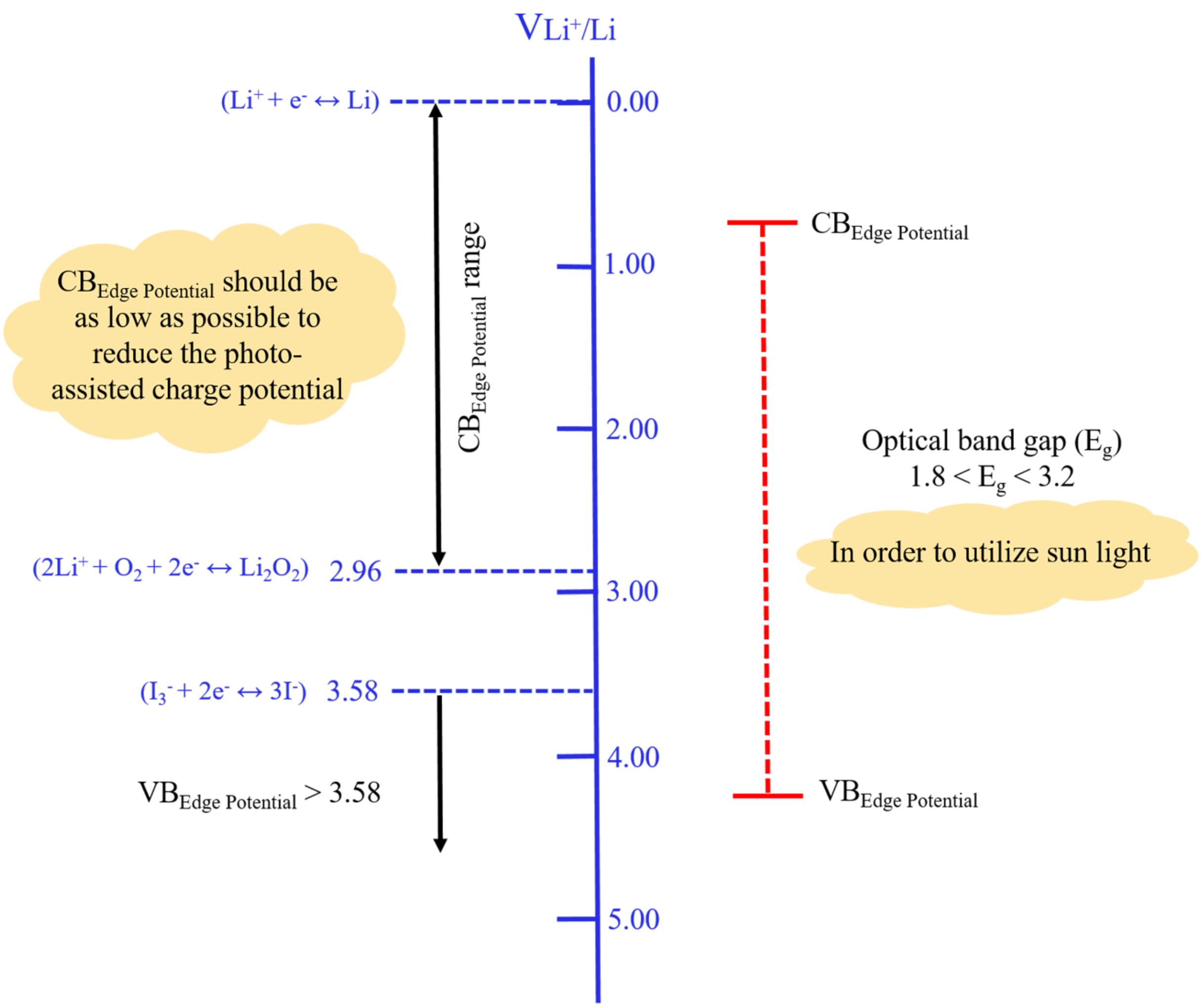

Figure S9. The tuning ranges for the CB and VB edge potentials to utilize the visible light in the photo-assisted charging of the Li-ion oxygen battery.

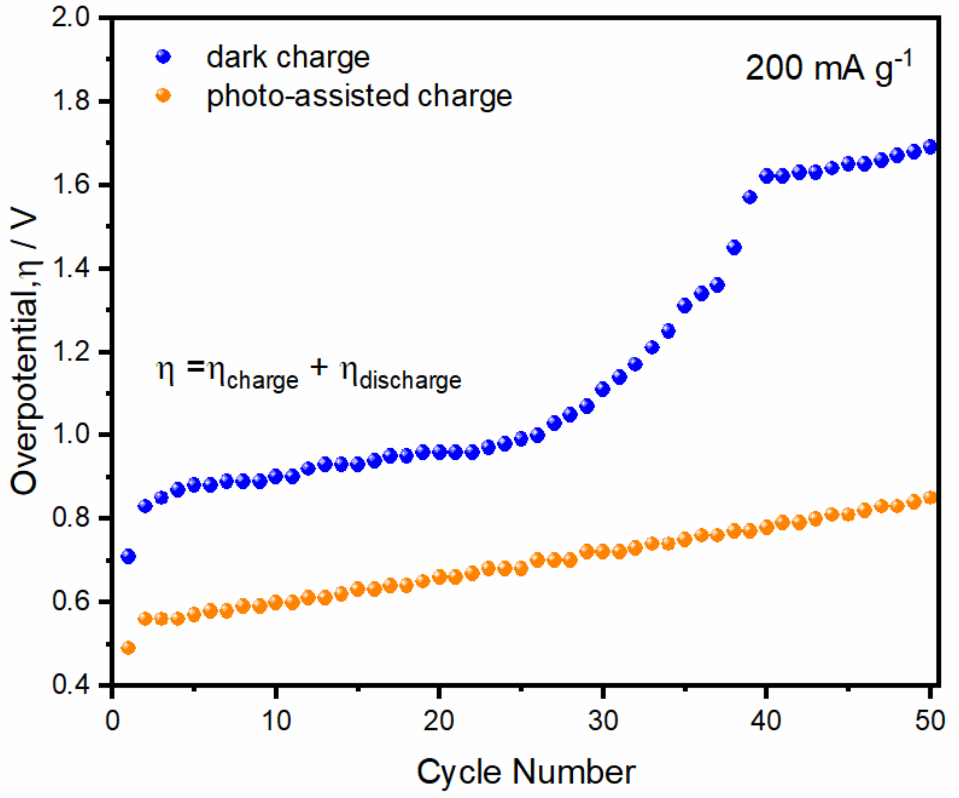

Figure S10. Total overpotentials (charge overpotential + discharge overpotential) at the dark-charge and photo-assisted-charge states (with pure g-C_3N_4 photo-catalyst) depend on the cycle number at 200 mA g^{-1} current density.

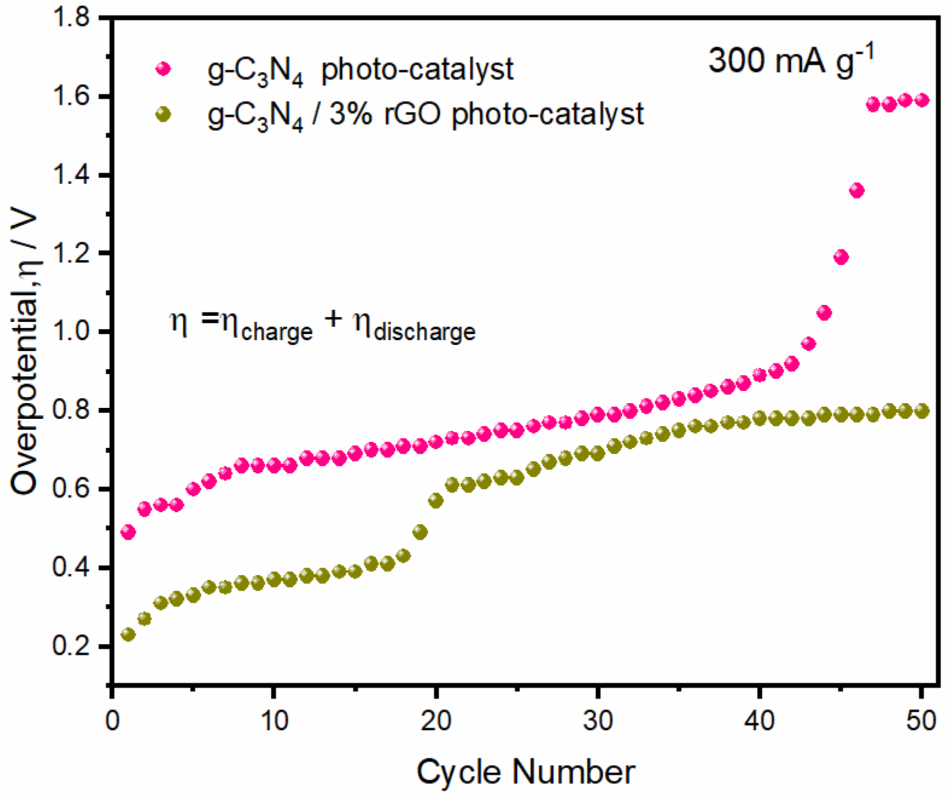

Figure S11. Total overpotentials (charge overpotential + discharge overpotential) at photo-assisted-charge states with the pure g-C_3N_4 and g-C_3N_4 / 3% rGO composite photo-catalysts depend on the cycle number at 300 mA g^{-1} current density.

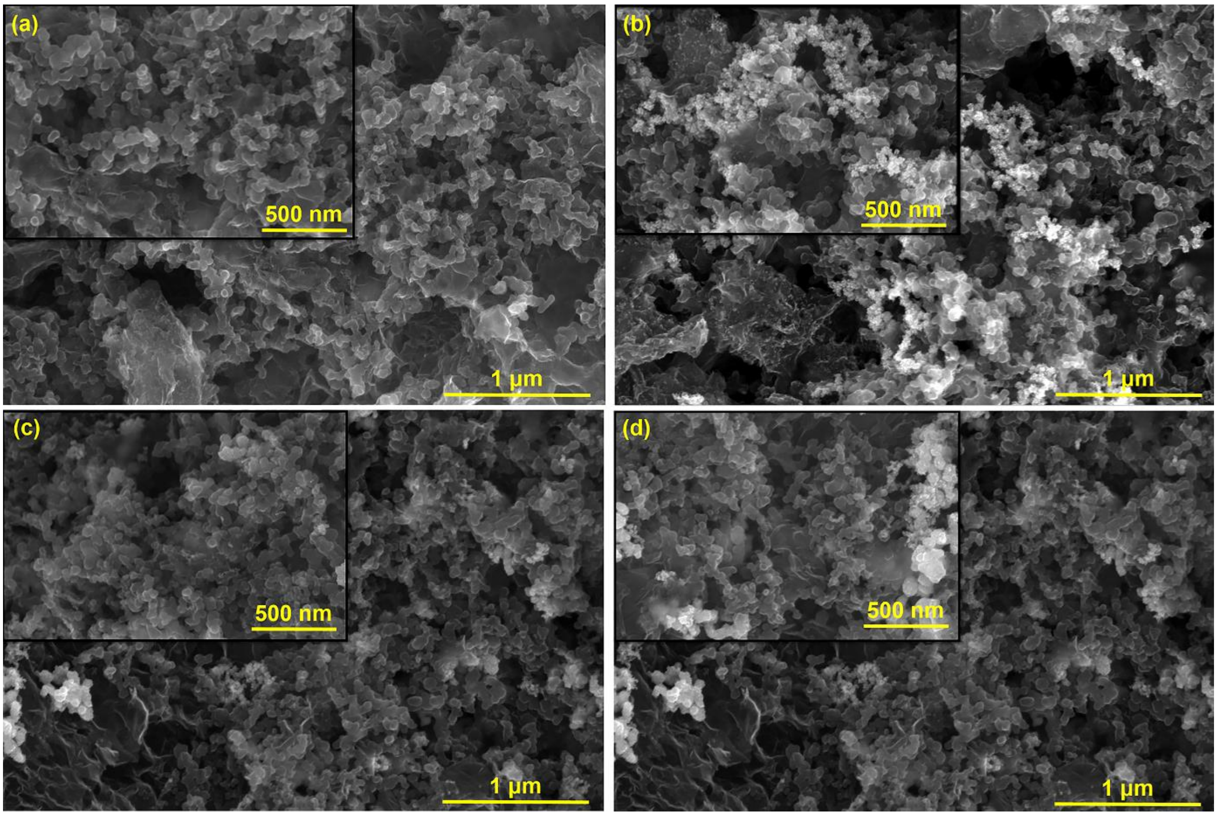

Figure S12. SEM images of (a) the as-prepared cathode, and the cathodes at (b) the discharged-state, (c) the dark-charged-state and (d) the photo-assisted-charged state.

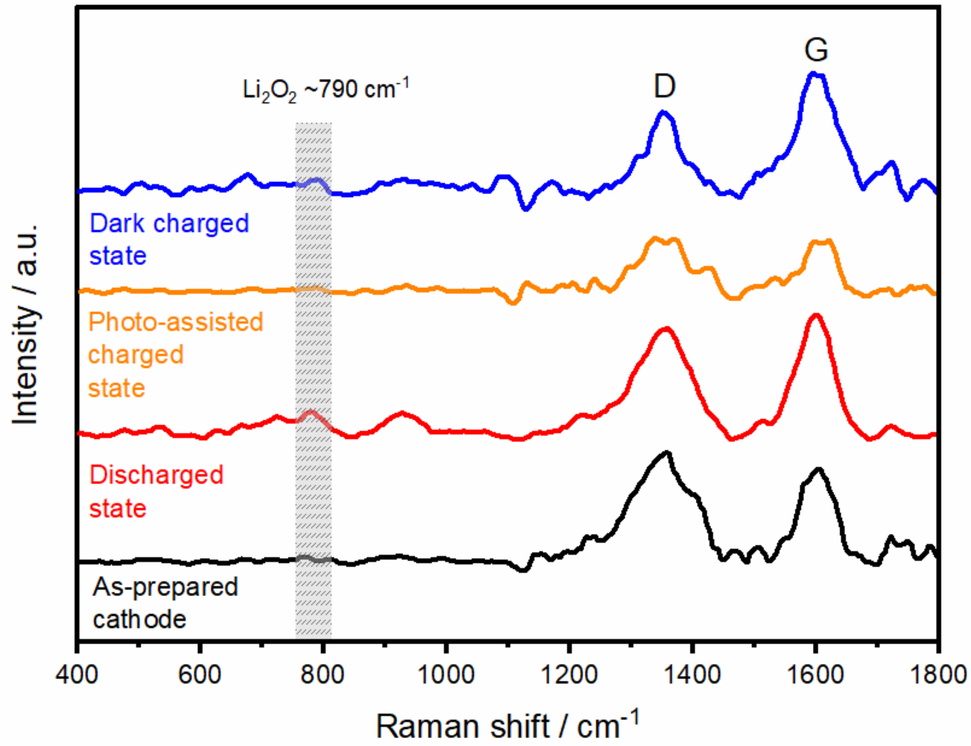

Figure S13. Raman spectra of the as-prepared cathode, and the cathodes at the discharged-state, the dark-charged-state and the photo-assisted-charged state.

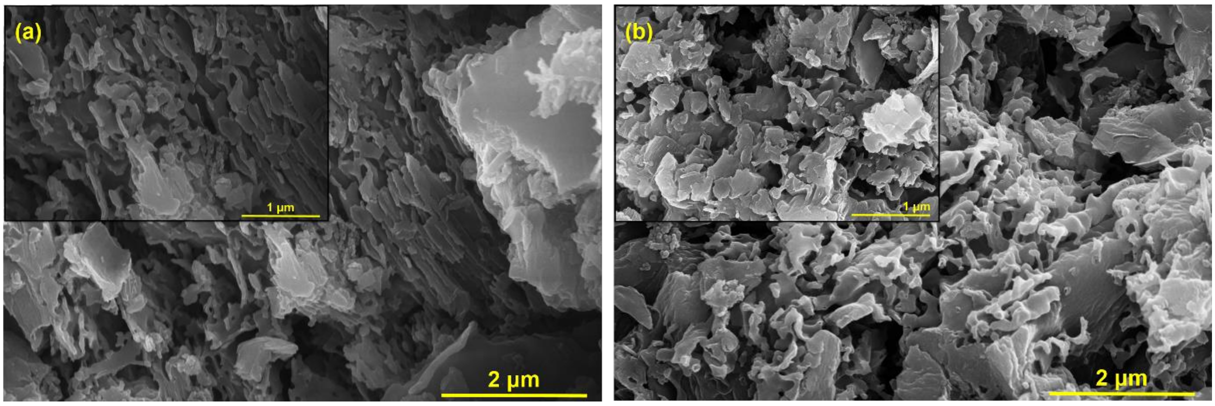

Figure S14. The SEM micrographs of the photo-electrodes at (a) the as-prepared condition and (b) the after 50-cycle constant capacity cyclic test.
